# Cancer and Health Inequalities

**DOI:** 10.1038/s41416-026-03599-4

**Published:** 2026-08-24

**Authors:** Donna Wakefield, Clare Bambra

**Affiliations:** 1 Population Health Sciences Institute, Faculty of Medical Sciences, Newcastle University, Newcastle upon Tyne, UK; 2 Wolfson Palliative Care Research Centre, Hull York Medical School, University of Hull, Kingston upon Hull, UK

**Keywords:** Cancer, Society

## Abstract

Cancer is subject to stark inequalities, with mortality rates 60% higher in the most deprived parts of the UK. Most research has focused on the role of behavioural risk factors or access to health care services. Insufficient attention has been paid to the influence of socio-economic factors across the whole cancer continuum. This essay argues that to reduce cancer inequalities and improve patient experience and clinical outcomes, we need to look more widely at inequalities in: (1) the risk of developing cancer; (2) the effectiveness of prevention, detection and diagnosis; (3) treatment; (4) survivorship, and (5) access to palliative care. We argue that a stronger awareness and understanding of the influence of the wider social, economic and cultural context of health can enable clinicians to improve the delivery of more equitable cancer care. We conclude by recommending ways forward to improve the cancer continuum for *everyone*.

## Introduction

Globally, one in five people will experience cancer in their lifetime, and it causes over 10 million deaths every year [1, 2]. In the UK, cancer accounts for approximately 170,000 deaths per year, and it affects almost 50% of people [3]. However, whilst cancer is increasingly a widespread human experience, it is also subject to stark socio-economic inequalities, not just in terms of unequal prevalence but across all aspects of the cancer continuum. This is a pressing health concern, and although there is a sizeable and growing literature, to date this has focused either on identifying epidemiological trends by social group; or eliciting the aetiological role of behavioural factors in relation to cancer risk (e.g. smoking or diet) or timely access to health care services [4, 5]. There has been far less attention (with some notable exceptions, such as the US Center for Population Health and Health Disparities framework [6, 7]) paid to the role of socio-economic factors in creating inequalities across the whole cancer continuum. Given the well-established importance of social inequality in the experience of most diseases and deaths, this absence within the cancer literature is surprising and significant [8]. Arguably, as a result, our understanding of—and ability to reduce—the unequal burden of cancer is severely restricted.

This short essay argues that to reduce socio-economic inequalities in cancer, we need to look *beyond behaviours* and at the wider social, economic and cultural context of health. It outlines and applies insights from the wider interdisciplinary health inequalities literature to the *whole* cancer continuum. It examines the influence of socio-economic status on: (1) the risk of developing cancer; (2) the effectiveness of prevention, detection and diagnosis; (3) treatment; (4) survivorship, and (5) palliative care. It argues that the prevention, treatment, experience and outcomes of cancer can be enhanced through a stronger awareness of the pervasive influence of social inequalities in health. It concludes by recommending how these insights can be applied to improve the cancer continuum for *everyone*.

## Social inequalities in health

Health inequalities are the systematic, avoidable differences in health which exist between different social groups (e.g. by socio-economic status, gender, ethnicity, place, disability, sexuality). They are socially produced, avoidable, and are therefore considered unfair and unjust [9]. The term health inequalities includes both inequalities in health outcomes (e.g. mortality rates, life expectancy, etc.) as well as inequalities in access to- and outcomes of- health care. Inequality is the preferred terminology in the UK and Europe and so we use it here. But in other contexts, slightly different terms are used. For example, in the USA and Canada, ‘health disparities’ is often employed, while for the World Health Organisation, the preferred term is ‘health inequities’ (avoidable differences in health between different social groups with different levels of need) [10].

Inequalities in health by socio-economic status (commonly measured as income, occupation or education) are not restricted to differences between the most privileged groups and the most disadvantaged: health inequalities exist across the entire socio-economic gradient [8]. Consistently, the finding has been that the lower the socio-economic position (e.g. income, education, occupation, deprivation), the worse the health, the higher the age-specific mortality rates and the shorter the life expectancy [8]. These socio-economic inequalities are experienced *intersectionally* with other axes of inequality [4] as people simultaneously belong to multiple social groups (e.g. they experience their socio-economic status, ethnicity, locality, gender, and sexuality simultaneously) [11]. This leads to complex experiences of social disadvantages and/or privileges which influence health in different ways.

These inequalities are very evident in cancer. In the UK, for example, cancer mortality rates are approximately 60% higher for people living in the most socio-economically deprived areas compared to the least deprived areas [12]. For all cancers combined this equates to 28,400 deprivation-associated cancer deaths per year [13]. This inequality in cancer survival due to deprivation is seen for many cancer sites, including bowel, breast and prostate cancer. It is particularly high – and widening – for lung cancer where mortality in the most deprived areas is almost three times higher than in the least deprived areas [12]. This cancer-deprivation gap (the term used to describe inequalities in cancer outcomes by deprivation) has also been demonstrated in many other countries (including Germany, Finland, Sweden, Norway, Denmark) [10, 14, 15]. Deprivation is an area-level measure of material circumstances, and not everyone living in a deprived area is necessarily subject to deprivation or poverty themselves. The association between cancer and lower socio-economic status though, is also evident in studies conducted with individual measures of education and income [16].

The causes of socio-economic inequalities in health are multifaceted. The scientific consensus is that they are a result of inequalities in the social determinants of health: the conditions in which we are born, grow, live, work and age [8]. They are the everyday conditions which influence our access to health-enhancing goods and services, and which limit our exposure to health-damaging risk factors [8]. The social determinants of health operate at different levels including: health-related practices (because health behaviours are shaped by the wider social, economic, cultural and policy context); social and community networks (resulting in psychosocial feelings of domination/subordination; superiority/inferiority, stigma or social in/exclusion); living and working conditions (including poverty, welfare and unemployment; work environment; housing; the environment; education; health and social care; transport) [8]; and economic, political and environmental conditions (macro-level structural determinants such as public policies, the organisation and performance of the economy, the role of the state in services and the labour market) [8]. Exposures to health-promoting, salutogenic (e.g. good quality, well-rewarded work), or health-damaging, pathogenic (e.g. damp, unaffordable housing) determinants are associated with social position (i.e our income, gender, ethnicity, etc.) [8]. Different social groups are unequally exposed to these health-damaging (down to the physiological and cellular level) or health-enhancing factors over their life course, as well as inter-generationally, resulting in inequalities. These vary across time and space leading to changing patterns of health inequalities [4].

## The unequal cancer continuum

The cancer continuum describes the various stages of the cancer journey across: (1) the risk of developing cancer; (2) the effectiveness of prevention, detection and diagnosis; (3) treatment; (4) survivorship, and (5) access to palliative care. This section outlines the socio-economic inequalities that exist across each of these different stages.

### Stage 1 - Unequal risk

The major environmental and behavioural risk factors for many cancers are shaped by social inequalities. For example, low-paid, low-skilled jobs are more likely to involve exposure to hazardous work environments in which carcinogens are present [17]. Likewise, air pollution rates are higher in lower-income or ethnic minority neighbourhoods [18]. Ultra-processed foods - are associated with higher cancer risk and mortality - are more affordable and more available in poorer areas: food poverty therefore increases cancer risk [19, 20]. Internationally, there is a social gradient for tobacco use, with those in lower socio-economic groups more likely to smoke; particularly increasing the incidence of lung and oral cancers [21, 22]. In England, around 24% of people aged 18 years and over living in the most deprived neighbourhoods are current smokers, compared to <7% living in the least deprived neighbourhoods [23]. Alcohol-related harm is also higher in poorer communities [24]. Many of these risk factors are also associated with stigma and blame—further exacerbating inequalities [25]. For example, smoking is widely portrayed as a personal ‘choice’—with the implication that those who develop lung cancer are less deserving of treatment [26]. This can lead to delays in accessing care by those affected [26].

### Stage 2 - Unequal prevention

People’s ability to reduce their cancer risk through lifestyle changes can be constrained by their socio-economic circumstances [27]. For example, physical activity is known to reduce cancer risk [28], but those in lower socio-economic positions face a multitude of barriers; such as material poverty (e.g. unable to afford the cost of gym subscriptions etc); time poverty (e.g. working long hours and/or informal care provision); or environmental poverty (e.g. living in poorer areas with high crime rates make physical activity feel unsafe). These factors result in those living in poverty being much less able to live healthy lives. Health promotion campaigns to raise awareness of cancer symptoms usually rely on high levels of literacy [29]. Those who have had less access to quality education are less likely to recognise concerning symptoms, resulting in delayed access to medical advice. These issues, as well as others such as social distrust, can also result in the unequal uptake of vaccination, screening and other detection services [30].

### Stage 3 - Unequal treatment

Socio-economic barriers lead to delayed diagnosis and inferior clinical pathways and outcomes. The inverse care law (whereby even in universal health care systems, lower-income communities have a higher need but less health care provision) reduces access to timely medical care appointments [31]. Many other barriers exist including challenges accessing affordable transport (especially in rural areas); the cost of attending appointments (in terms of lost wages as well as sundries such as food); or navigating complex referral and diagnostic systems [32, 33]. These can all result in delayed or missed appointments and presentation at a later cancer stage. Once in the system, people from lower socioeconomic backgrounds are significantly less likely to receive advanced treatments such as surgery, chemotherapy or novel biological/targeted treatments [34, 35]. There are also inequalities in treatment adherence.

### Stage 4 - Unequal survivorship

Socio-economic inequalities impact on survivorship and survivors from lower socio-economic groups are less likely to live well beyond cancer; experiencing a lower quality of life post-treatment [36]. They are more likely to experience late treatment effects, and they have an increased likelihood of long-term physical and/or psychological effects of their cancer. Survivors who experience late effects are more likely to remain out of work and experience income loss, further impacting on their wellbeing [37]. Many countries provide financial support to those who have lost earnings due to poor health, however accessing these systems can be challenging with patients unaware or otherwise missing out on entitlements [38]. The monetary impact of cancer is not usually screened for by healthcare professionals and so signposting to welfare/financial support services cannot be undertaken.

### Stage 5 - Unequal care

Palliative care is an approach that improves the quality of life of patients and their families, diagnosed with incurable illness, including cancer. It relieves suffering through identification, assessment and treatment of physical, psychosocial and spiritual problems and enables people to die with comfort and dignity [39]. Those experiencing socio-economic deprivation often have a higher symptom burden (for example, worse pain [40]) and so have the most need for specialist palliative care support, yet they are less likely to receive it [41, 42]. As a result, they have a lower quality end-of-life experience, with more frequent emergency department visits and hospital admissions [43]. People living in poverty are significantly more likely to die in hospital, compared to their home or a hospice [43]. They and their families often face financial problems and debt related to affording suitable housing, food, heating, or electricity and funeral costs [44, 45]. This ‘financial pain’ was first noted by Dame Cicely Saunders, founder of the modern hospice movement, whose concept of ‘total pain’ acknowledged that financial suffering and social struggles contribute to patient distress [46]. However, few UK doctors have any personal or family experience of such financial pain as only around 5% of them come from the most socio-economically deprived backgrounds [47]. The additional burdens of poverty faced by their cancer patients may remain unexplored, with a lack of assessment and signposting to sources of financial support [46, 47].

## Conclusion: Towards more equal cancer care

Whilst there has been much improvement in outcomes over the last few decades across all stages of the cancer continuum, systematic inequalities have persisted. This essay has outlined some of the ways in which the socio-economic inequality impacts patient experiences across the whole continuum, resulting in a highly unequal cancer morbidity and mortality burden. Reducing cancer inequalities requires recognition that health is shaped not only by individual choices, but by the conditions in which people live and work. This means action on the economy, on decent housing, and on cleaner environments, and creating the societal conditions in which everyone can live a healthier life. However, understanding the social context of cancer can also enable clinicians to improve the design and delivery of more equitable cancer care. There are examples of how disadvantaged communities have come together to provide peer support; for example, Compassionate Communities is an international movement of community support around death, dying and grief [48]. There is also evidence that inequalities in the continuum can be reduced through delivering screening and diagnostic services in more accessible community settings [49–51]; poverty-proofing cancer care; providing financial screening and referral to welfare services as a routine part of care provision [52, 53]; and collecting information about the social determinants of health as part of clinical research and considered in design and evaluation [54]. As such, getting *beyond behaviours* in our understanding of inequalities can improve the cancer continuum for *everyone*.
